# 

**Published:** 1841-01-01

**Authors:** 


### MEDICO-CHIRURGICAL REVIEW,

AND

### JOURNAL

OF

### PRACTICAL MEDICINE.

*(NEW SERIES.)*

VOLUME THIRTY-FOUR,

[1st of OCTOBER, 1840, to 31st of MARCH,]

1841.

VOL. XIV. of DECENNIAL SERIES.

EDITED

By JAMES JOHNSON, M.D.

PHYSICIAN EXTRAORDINARY TO THE LATE KING,

AND

HENRY JAMES JOHNSON, Esq.

LECTURER ON ANATOMY AT THE SCHOOL OF ST. GEORGE'S HOSPITAL IN KINNERTON STREET.

Surgeon Genl's Office. LIBRARY. 57474 Washington, D.C.

LONDON:

PUBLISHED BY S. HIGHLEY, 32, FLEET STREET,

*Re-printed in New York, by Mr. Wood.*

1841.

WI
ME 75 T

PRINTED BY F. HAYDEN,
Little College Street, Westminster.

### INDEX.

A.

Abdominal tumors, Dr. Bright on.... 258
Ablation of the clavicle............ 228
Abscess over the lachrymal sac...... 46
Accessory nerves, Arnold on the .... 495
Accumulations in the colon ........ 260
Aconite, poisoning by ............ 498
Acupuncturation, neuralgia treated by 501
Acute inflammations, opium in...... 570
Affections of the cornea .......... 37
Albuminous assimilation .......... 444
Albuminous urine ................ 448
Alcock on injuries of joints ........ 314
Alleged dislocation of its axis ...... 238
Alterations of the blood in different diseases .................... 485
Alum, treatment of gonorrhœa with.. 514
Amaurosis, partial ............... 275
Amenorrhœa .................... 82
Ammonia in diabetes.............. 254
Amputations in Paris.............. 476
Amputations, want of success after .. 477
Amputations, Blandin's....... .... 478
Anæsthesia of the trigeminal nerve .. 230
Anatomy, Monro's lectures on ...... 404
Anatomy of the nerves of the uterus by Dr. Lee ................... 479
Anchyloses, false ................ 222
Anchylosis, barbarous operation for . 185
Ancle-joint, dislocation of the ...... 296
Andral on endocarditis ............ 194
Andral on carditis ................ 195
Andral on the changes of the blood in disease........................ 196
Aneurysm of carotid .............. 227
Aneurysm, varicose, in the neck.... 228
Aneurysm of the innominata ...... 339
Aneurysm, Horner's cases of ...... 563
Aneurysms, Mr. Thurnam on ...... 326
Aneurysms, liability of sinuses to.... 326
Aneurysms of the ascending aorta .. 335
Annual report of the registrar general 115
Anus, treatment of fissures of the .. 225
Aorta, obliteration of the .......... 233
Aphorisms of practical surgery...... 514
Aqueous assimilation.............. 423
Arnold on the pneumogastric and accessory nerves ................ 495
Arnott's case of osseous tumor of the uterus ...................... 309
Arnott on stricture of the urethra .. 465
Arteria innominata, aneurysm of the 339
Articular rheumatism, Bouillaud on.. 65
Articulations, classification of wounds of............................ 321
Artificial labour, inducement of .... 525
Ascending aorta, aneurysms of the .. 335
Ashwell on diseases of women...... 78
Assimilation, process of............ 157
Assimilation, secondary............ 160
Auscultation, disparagement of .... 214
Auscultation, Beau on ............ 488
Auditory organs in a deaf and dumb person ...................... 229

B.

Barlow on diabetes .............. 253
Beau's sounds of respiration........ 208
Beau on auscultation.............. 488
Belladonna in epilepsy ............ 499
Births, deaths, and marriages in England ...................... 135
Blacklock on hydrocephalus ........ 300
Blandin on erysipelas.............. 181
Bleeding in mania ................ 568
Blindness produced by onanism .... 575
Blindness produced by venery ...... 575
Blood corpuscules, Gulliver on ...... 6
Blood, changes of the, in disease .... 196
Blood, alterations of the............ 485
Bloodletting in hydrocephalus ...... 300
Bloxam on the placenta............ 313
Bougie, dangers of the ........ ... 455
Bouillaud on articular rheumatism .. 65
Brandy and salt, death from ........ 574
Brettoneau on fissures of the anus .. 225
Bright on abdominal tumors........ 258
Browne on luxation of the humerus.. 567
Browne on bleeding in mania ...... 568
Budd on emphysema of the lungs .. 9
Burton on the effects of lead on the gums ........................ 13

C.

Cachexia, organization of lymph in.. 310
Calcutta, topography and climate of.. 97
Cancer, origin of, in the veins ...... 206
Cancer of the upper lip ............ 454
Cancer of the stomach ............ 455
Cane's cases of amaurosis .......... 575
Carotid, aneurysm of.............. 227
Cases in surgery—Syme's .......... 533

Catalogue of Irish College of Surgeons 453
Catheter, mode of introducing in difficases .......................... 584
Cervical vertebra, dislocation of a .. 238
Cervix uteri, ulceration of the ...... 225
Chest, deformity of the ....... .... 463
Chest, fatal wound of the .......... 470
Chlorosis....... ................. 78
Cholera ........................ 103
Cholera, treatment of ............ 105
Chorea, fatal case of .............. 539
Christison on opium in acute inflammation ........................ 570
Classification of wounds of the articulations......................... 321
Clavicle, ablation of the............ 228
Climate, influence of .............. 147
Clinical report, Lendrick's ........ 546
Clinique, surgical, Lisfranc's......... 220
Common iliac, successful ligature of 226
Cooper's guide to Madeira.......... 176
Cooper on stricture of urethra ...... 245
Cooper on the cure of inguinal hernia 257
Cooper on hydrocele ............... 540
Cooper, Sir Astley, death of........ 591
Cornea, affections of the .......... 37
Coulson on diseases of the hip-joint 177
Cracked tongue .................. 581
Cure of hernia, new method for .... 239
Cure of squinting, Guthrie on the .. 241
Cure of inguinal hernia............ 257
Curling on a rare species of hydatid.. 336
Cutaneous scrofula, Lugol on ...... 508
Cut-throat, case of recovery from.... 312
Cystic oxide, Jones on sulphur in.... 34

D.

Dalrymple on the rapid organization of lymph.............. ...... ... 310
Deaf and dumb person, auditory organs in a...................... 229
Deafness, new treatment of ........ 503
Death from brandy and salt ........ 574
Death of Sir Astley Cooper ........ 591
Deformity of the chest ............. 463
Diabetes, Mr. Barlow on .......... 253
Diabetes ........................ 428
Diabetic diuresis in young children .. 434
Dickson's case of wound of the intestine .......................... 301
Dieffenbach's operation for stammering 552
Digitalis in epilepsy, Sharkey on .... 357
Disease of the spinal cord, Stanley on 16
Diseases of the eye, Tyrrell on...... 35
Diseases of the town and open country 144
Diseases induced by mercury........ 510
Dislocation of a cervical vertebra.... 238
Dislocation of the ancle-joint ...... 296
Disparagement of auscultation, by M. Lugol ........................ 214
Distortions, Ward on ............ 461
Division of the sphincter ani ...... 282
Domestic management of the sickroom 594
Douglas on dislocation of the ancle-joint.......................... 296
Dupuytren's surgical aphorisms .... 514
Dysentery ...................... 108
Dysmenorrhea .................. 87

E.

Echinococcus hominis ............ 336
Efficacy of digitalis in epilepsy ...... 357
Egypt & Mohammed Ali—Dr.Madden 593
Electrical actions in living bodies .... 492
Emetics in phthisis................ 249
Emmenagogues ... .............. 85
Emphysema of the lungs, Dr. Budd on 9
Emphysema, spontaneous. ........ 509
Emphysema of the lungs, symptoms of 583
Endo-pericarditis after rheumatism .. 193
Endo-carditis, Andral on .......... 195
England, births, deaths and marriages in .......................... 135
Epidemic ophthalmia............ .. 545
Epidemics, progress of ............ 149
Epidemics of small-pox. .......... 149
Epilepsy, Sharkey on digitalis in .... 357
Epilepsy, belladonna in .... ....... 499
Erectile tumor of the tongue......... 224
Excision of the head of the femur .. 314
Excision of the head of the humerus 314
Excision of the knee-joint.......... 315
Excision of the elbow joint ........ 315
Excision of the wrist .............. 315
Exercise, retardation of the pulse by 464
Exostosis on a phalanx ............ 282
Extirpation of fifth metacarpal bone 468

F.

Face of a man eaten by a pig........ 453
Fever, Hudson on the poison of .... 1
Fever, infectious animal poison of.... 2
Fever, sources of ........... .... 2
Fever, nature of the poison of ...... 8
Fever, mode of action of the poison of 13
Fever, theory of ................ 13
Fever, mortality of................ 12
Fever, origin of .................. 14
Fever, continued, infection of ...... 16
Fistula urethræ, autoplastic operation for ......................... 475
Fistulæ, Lisfranc on .............. 517
Flat bones, union of fractures of .... 337
Fore-arm, wound of the .......... 469
Fracture apparatus, immoveable .... 573
Fractures of flat bones, union of .... 337
Fractures of the clavicle .......... 473
France, progress of medicine in.. ... 530
Freeman on valvular disease of heart 294
French, barbarity of the............ 185
French official document on re-vaccination ................. ... 528

Functions of the par vagum ........ 495
Functions of the spinal accessory.... 495

G.

Gangrena senilis.................. 549
Gangrene, hospitals in Paris........ 240
Gannal's new process of embalming.. 402
Gas-baths ..... ................. 457
Gastromalacia, Winter on.......... 217
Gastromalacia, anatomical characters of............... .... ...... 218
Gastromalacia, causes of .......... 219
Gastromalacia, treatment of.. ..... 220
Gendrin's traité philosophique...... 53
Gestation, protracted.............. 556
Glasgow phrenological association .. 289
Gonorrhœa, seat of, in women...... 207
Gonorrhœa, seat of, in women...... 512
Gonorrhœa, treatment of ......... 514
Graves on the treatment of various diseases ..................... 577
Green on medical reform .......... 414
Gregory, Dr. v. variolæ vaccinæ .... 582
Gulliver on blood corpuscules ...... 6
Gums, effect of lead on the ........ 13
Guthrie on the cure of squinting .... 241
Guy's Hospital Reports............. 244

H.

Hæmatocele, operations for ........ 472
Hæmorrhage, sources of, after lithotomy ........................ 558
Hall on the pathology of the nervous system .................. 25 and 32
Hamerton on syphilis ............. 580
Hawes' medical reform bill ......... 186
Hawkins on foreign bodies in the larynx 21
Head, injuries of the.............. 277
Heart, Paget on white spots on the.. 7
Heart, valvular disease of the ...... 293
Hepatic tumors ................... 270
Hernia, new cure of ............. 239
Hernia, inguinal, cure of .......... 257
Herniæ, radical cure of............ 523
Hip-joint, Coulson on diseases of the, 177
Hip-joint, congenital displacement of, 179
History of new operation for squinting 518
Horner's cases of aneurysm ........ 563
Hospital gangrene in Paris ........ 240
Houston's catalogue of Irish College of Surgeons..................... 453
Hudson on the poison of fever...... 1
Hughes on incipient phthisis.. ..... 248
Humerus, luxation of the .......... 567
Hydatid, Curling on a rare species of, 336
Hydrocele, Cooper on ............. 540
Hydrocephalus, bloodletting in...... 300

I.

Immobility of the lower jaw. ...... 281
Immoveable fracture apparatus...... 573
Inability to distinguish colours...... 275
Incipient phthisis, Dr. Hughes on ... 248
Inducement of artificial labour ...... 525
Inflammation of the cervix uteri .... 95
Influence of forms of ships on health, 132
Infra-orbital nerve, injury of the.... 468
Inguinal hernia, cure of............ 257
Inguinal hernia, Morton on ........ 465
Injuries of the head .............. 277
Injuries of the spine .............. 279
Injuries of joints, Mr. Alcock on .... 314
Inoculation after vaccination........ 579
Insanity, Leuret on ............... 343
Intermittent fever ................ 105
Intermittent fever, pathology of..... 206
Intestine, fatal wound of the........ 301
Iris, inflammation of the ........... 44
Irish College of Surgeons, catalogue of 453
Ironmonger, stomach of a.......... 454

J.

Johnson's nuces philosophicæ ...... 456
Joints, Alcock on injuries of........ 314
Jones on sulphur in cystic oxide .... 34

L.

Lactic acid ...................... 437
Laryngismus stridulus ............ 550
Larynx, Hawkins on foreign bodies in the........................ 21
Lead, effects of, on the gums........ 13
Lectures on anatomy. By Dr. Monro 404
Lee's nerves of the uterus ... ...... 479
Leg, compound fracture of the...... 224
Lendrick's clinical report .......... 546
Leucorrhœa ...................... 91
Leuret on belladonna in epilepsy .... 499
Leuret on Insanity............. .. 343
Lever's case of tumor in the pelvis ... 342
Lice, co-existence of, with scrofula... 509
Ligature of common iliac, successful, 226
Lisfranc's surgical clinique ......... 220
Lisfranc's surgical clinique .......... 517
Liston on vessels in granulation .... 18
Lithotomy, sources of hæmorrhage after.......................... 558
Liver, encysted tumor of the.... ... 230
Liver, tumors of the .............. 259
Liver, enlargements of the.......... 265
Liver, fatty change in the .......... 268
Living bodies, electrical actions in ... 492
Lower jaw, immobility of the ...... 281
Lugol's disparagement of auscultation 214
Lugol on cutaneous scrofula........ 509
Lungs, Dr. Budd on emphysema of.. 9
Luxation of the humerus .......... 567

M.

Madden, Dr. on Egypt & Mohammed Ali 593
Madeira, Cooper's guide to ......... 176
Males and females, diseases of ...... 141

Malta, Dr. Sankey on .............. 389
Mamma, engorgement of the, in a man 221
Man, menstrual flux in a .......... 229
Man, face of a, eaten by a pig ...... 453
Mania, Browne on bleeding in ...... 568
Mare, nerves of the uterus in the.... 483
Markham's surgical practice of Paris, 180
Martin on climate of Calcutta ...... 97
Matter, escape of, through muscles .. 471
Meade on the union of fractures of flat bones .................... 337
Medical statistics, naval............. 115
Medical reform bill.................. 186
Medical Reform Bill, Warburton's ... 283
Medical reform .................... 288
Medical and physiological commentaries. By Dr. Payne.............. 392
Medical reform, touchstone of ...... 414
Medical reform .................... 585
Medicine pratique, Traité philosophique de ..................... 53
Medicine, progress of, in France .... 530
Medicinische Jahrbucher .......... 229
Medico-chirurgical Transactions, Vol. XXIII........................ .. 1
Medico-chirurgical Transactions .... 309
Menorrhagia ....................... 89
Menstrual flux in a man ........... 229
Menstruation, decline of............ 95
Mercury in small-pox .............. 507
Mercury, diseases induced by ....... 510
Monro's lectures on anatomy........ 404
Morton on inguinal hernia ......... 465
Motory and sensory nerves, &c. ..... 498
Mud-baths ......................... 457
Muscles, subcutaneous section of.... 226

N.

Naval medical statistics........ .... 115
Navy, health of the................. 115
Nerves of the uterus, Dr. Lee's...... 479
Nerves of the gravid uterus ........ 479
Nerves of the gravid uterus, 9th month 481
Nerves of the gravid uterus, 4th month 482
Nerves of the uterus after delivery .. 483
Nerves of the uterus in the mare.... 483
Nervous system, Hall's pathology of the ........................ 25, 32
Neuralgia treated with acupuncturation 501
Neuralgia, sacro-lumbar ........... 501
Neuralgia, severe brachial .......... 501
Neuralgia, lumbo-sciatic .......... 502
New York Hospital, report from .... 277
New process of embalming. By M. Gannal ......................... 402
New mode of inducing artificial labour 525
Nuces philosophicæ, Johnson's...... 456
Nux vomica, in vomiting .......... 230

O.

Obliteration of the aorta ........... 233
Observations on squinting ......... 521
Œsophagus, perforation of the, by a bougie ......................... 454
Onanism, night-blindness from. .... 575
Operations for hæmatocele........... 472
Operations for stammering ......... 551
Ophthalmia, epidemic .............. 545
Opium in acute inflammations ...... 570
Orfila on poisoning................. 217
Organization of lymph in cachexia .. 310
Origin of cancer in the veins........ 206
Origin of tubercles.................. 214
Osseous tumor of the uterus......... 309
Oxalic acid diathesis .............. 435

P.

Paget on white spots on the heart ... 7
Palpebræ, tumors over the.......... 47
Paraplegia ... ............... 178, 423
Paris, Markham's surgical practice of, 180
Paris, erysipelas prevalent in........ 181
Paris, hospital gangrene in.......... 241
Paris, surgery in .................. 467
Paris, amputations in .............. 476
Pathology of the nervous system .... 25
Pathology of typhus fever, Rostan on, 200
Pathology of intermittent fever...... 206
Payne's medical & physiological commentaries ...................... 392
Pelvis, case of tumor in the ........ 342
Penetrating ulcer of the stomach .... 235
Peritoneum, malignant disease of.... 261
Petrequin's treatment of deafness.... 503
Phagedenic ulcers ................. 221
Phalanx, exostosis on a............ 282
Phrenological association at Glasgow, 289
Phthisis, incipient, Dr. Hughes on .. 248
Phymosis, operation for............ 474
Physiology and hygiene, Reveillé-Parissæ on .............. ........ 360
Placenta, Mr. Bloxam on the ....... 313
Pneumo-gastric nerves, functions of the ............................ 495
Poem on syphilis .................. 234
Poison of fever, Dr. Hudson on the .. 1
Poisoning, Orfila on ............... 216
Poisoning from aconite ............ 498
Polypus of the uterus..... ......... 575
Pott's gangrene ................... 549
Practical surgery, aphorisms of...... 514
Progress of medicine in France...... 530
Protracted gestation ............... 556
Prout on stomach and urinary diseases 153
Pulse, retardation of the, by exercise, 464

R.

Radical cure of herniæ ............ 523
Recovery from cut-throat .......... 310
Rectum, contraction of the ........ 223
Rectum, ulcer of the ............... 282
Registrar-general, annual report of .. 115

Remedies against sleeplessness...... 504
Respiratory sounds, Beau on the .... 208
Results of re-vaccination .......... 527
Re-vaccination, results of .......... 527
Re-vaccination, French official document on .................... 528
Reveillé-Parissæ on physiology and hygiene .......................... 360
Rhatany injections in fissures of the anus.......................... 225
Rheumatism, articular, Bouillaud on, 65
Rheumatism, endo-pericarditis after.. 193
Robertson on spinal diseases........ 385
Rostan on the pathology of typhus fever ........................ 200
Rostan on the treatment of typhus fever ........................ 203
Roux' reprehensible operations...... 467

S.

Saccharine assimilation............ 425
Sankey on Malta ................ 389
Sciatica ........................ 177
Scirrhus of the stomach .......... 567
Sclerotitis ...................... 43
Seasons, influence of .............. 147
Sensory and motory nerves ........ 498
Serous urine .................... 449
Sharkey on digitalis in epilepsy...... 357
Ships, influence of forms of, on health 132
Sick room, domestic management of.. 594
Sinuses, liability of, to aneurysm.... 326
Skey on the venereal disease........ 355
Sleeplessness, remedies against...... 504
Small-pox, mercury in ............ 507
Softening of the stomach .......... 217
Sounds of respiration, Beau on .... 208
Speculum, Dr. Markham on the .... 475
Spence on the sources of hæmorrhage after lithotomy ................ 558
Sphincter ani, division of the ...... 282
Spinal cord, Stanley on disease of.... 16
Spinal cord, morbid reflex action of.. 25
Spinal affections, Dr. Robertson on.. 385
Spine, injuries of the.............. 279
Spine, Ward on distortion of the.... 461
Spontaneous hydrophobia .......... 231
Spontaneous combustion .......... 235
Spontaneous emphysema .. ........ 509
Squinting, Guthrie on the cure of.... 241
Squinting, history of a new operation for .......................... 518
Squinting, observations on ........ 521
Stafford's case of recovery from cut-throat ........................ 312
Stanley on disease of spinal cord .. . 16
Stammering, operations for ....... 551
Stammering, Dieffenbach's operation for .......................... 552
Starch bandages ... ............. 473
Stomach, softening of the.......... 217
Stomach and urinary diseases, Prout on ...................... 153 423
Stomach, penetrating ulcer of the .. 235
Stomach and brain, Dr. Wightman on the sympathetic relations between 410
Stomach of an ironmonger ........ 455
Stomach, scirrhus of the .......... 567
Strangulated hernia, Travers' case of 2
Stricture of the urethra, Cooper on.. 245
Stricture of the urethra, treatment of 243
Stricture of the urethra, caustic in .. 247
Stricture of the urethra, Dr. Arnott on 465
Structure of the human placenta.... 313
Subcutaneous section of muscles.... 226
Successful ligature of common iliac.. 226
Sulphur in cystic oxide, Jones on.... 34
Surgery in Paris .................. 467
Surgery, Syme's cases in .......... 533
Surgical practice of Paris .......... 180
Surgical clinique, Lisfranc's ........ 220
Surgical clinique, Lisfranc's ........ 517
Surgical cases, Dr. Watson's.... ... 543
Syme's cases in surgery............ 533
Symptoms of emphysema of lungs.... 583
Syphilis, Hamerton on ............ 580
Syphilis, a poem on ............. 234

T.

Teeth, looseness of the ............ 577
Thornton's case of abdominal tumor 298
Thurnam on aneurysms............ 326
Tinea capitis .................... 577
Topography and climate of Calcutta 97
Touchstone of medical reform ...... 415
Tracheotomy, Travers on .......... 23
Traité philosophique de Medecine Pratique .......................... 53
Traitement moral de la Folie. Par M. Leuret ...... ................ 343
Travers' case of strangulated hernia.. 2
Travers on tracheotomy............ 23
Trigeminal nerve, anæsthesia of the.. 230
Tubercles, origin of................ 214
Tumor in the pelvis .............. 342
Typhus fever, Rostan on the pathology of.............................. 200
Typhus fever, Rostan on the treatment of ...................... 203
Tyrrell on diseases of the eye.... ... 35

U.

Ulcer of the stomach, perforating.... 235
Ulcer of the stomach, symptoms of.. 237
Ulcer of the stomach, treatment of .. 237
Urea, excess or deficiency of, in the urine .......................... 444
Urethra, Cooper on stricture of the.. 245
Urethra, Dr. Arnott on stricture of .. 465
Uterine plexuses of nerves.......... 480
Uterus, osseous tumor of the ...... 309
Uterus, Dr. Lee's nerves of the .... 479

Uterus, gravid, nerves of the........ 479
Uterus, polypus of the ............ 575
Uvulatome ..................... 574

V.

Vaccination, inoculation after ...... 579
Valvular disease of the heart ...... 293
Varicose aneurysm in the neck. .... 228
Varicose aneurysm................ 327
Veins, origin of cancer in the ...... 206
Velpeau on the publishing of cases .. 467
Venereal disease, Skey on the ...... 355
Veratrine, on the use of .......... 232
Vessels in granulation, Liston on.... 18
Vibratory râles .................. 491
Vicarious menstruation........... 86
Volition, distinct influence of ...... 32
Vomiting, nux vomica in .......... 231

W.

Waller on diseases of the womb .... 49
Warburton's medical reform bill .... 283
Ward on distortions of the spine.... 461
Watson's surgical cases ........... 543
Westminster Ophthalmic Hospital .. 241
White spots on the heart, Paget on.. 7
White swelling ............ ..... 222
Wickham on aneurysm of the innominata.......................... 339
Wightman on the sympathetic relation between the stomach and brain 410
Williamson on scirrhus of the stomach 567
Wills' Hospital, report from........ 275
Winter on gastromalacia .......... 217
Womb, Waller on diseases of the.... 49
Women, Ashwell on diseases of...... 78
Women, seat of gonorrhœa in ...... 207
Women, seat of gonorrhœa in ...... 512
Wound of the intestine ......... 301

### Notices to Correspondents.

The Sequel of Dr. Hudson's valuable Paper on "Fever" will be concluded in our next Number.

Mr. Ewart's Paper on the "Medical Topography of Alston Moor," has been unavoidably postponed till our July Number.

We have received "Illustrations of the Arteries connected with Aneurysm and Surgical Operations. By G. D. Dermott." This very useful work is now republished by Mr. Hill at the unusually small price of *one guinea!* A valuable boon to students and to surgeons who have not the advantage of constant reference to the dissecting room.

The Anatomy of the Arteries of the Human Body, by Richard Quain, is a work which we cannot praise too highly. Every public professional body should subscribe liberally for it, and every surgeon who can afford it should possess it.

Dr. Willis's Illustrations of Cutaneous Disease are completed. Dr. Anthony Todd Thompson's are in progress. We shall revert, in our next, to both, recommending, in the present instance, each.

Dr. Johnson's work on the "Stream of Human Life," has been translated into the German language, by Dr. Calmann, of Leipzig. We thank Dr. C. for the German copy.

### BIBLIOGRAPHICAL RECORD.

1. A Practical Treatise on the Function and Diseases of the Unimpregnated Womb. Illustrated by Plates, &c. With a Chapter on Leucorrhœa, Fluor Albus, or "Weakness." By CHARLES WALLER, M.D. Consulting Physician-Accoucheur to the London Midwifery Institution, &c. Octavo, pp. 200. Churchill, October, 1840.

---

2. The Naturalist's Library. Conducted by Sir W. JARDINE, Bart. F.R.S. &c. &c. Ichthyology. Vol. II., treating on the Nature, Structure, and Economical Uses of Fishes. By J. S. BUSHNAN, M.D. F.R.S.E., &c. Octavo, pp. 219. With numerous coloured Plates. Lizars, Edinb. Highley, London. July, 1840.

---

3. The Naturalist's Library. Conducted by Sir WILLIAM JARDINE, Bart. &c. Introduction to Entomology, Vol. I., by JAS. DUNCAN, M.W.S. Octavo, pp. 230, with numerous coloured plates. Edinb. Lizars; London, Highley, Fleet Street; and Curry, Jun. Dublin. September, 1840.

---

4. Second Annual Report of the Registrar-general of Births, Deaths, and Marriages, in England, with Appendices. Presented to both Houses of Parliament. 4to. Sept. 1840.

---

5. On the Nature and Treatment of Stomach and Urinary Diseases: being an Inquiry into the Connexion of Diabetes, Calcalus, and other Affections of the Kidney and Bladder, with Indigestion. By WILLIAM PROUT, M.D. F.R.C. Physicians. Octavo, pp. 484. Third Edition. Churchill, London. October, 1840.

---

6. The Cyclopædia of Practical Surgery, embracing a complete view of all the Departments in Operative Medicine. Edited by W. B. COSTELLO, M.D. &c. Part VII. October, 1840.

☞ *This part contains, Cancer, by Dr. Walshe—Cancrum Oris, by P. Henry Green, M.B.—Castration, by Sir Astley Cooper, Bt. —Cataract, by Dr. Watson—Catheter, by Dr. Costello—Caustics and Cauterization, by Dr. A. Ure—Cephalæmatoma, by Dr. Walshe—Cephalotomy, by Dr. Burges—Chondritis, by T. S. Wells, Esq.*

7. Derangements of the Digestive Organs, (primary and reflex.) By ROBERT DICK, M.D. Author of a Treatise on Diet and Regimen. Octavo, pp. 384. Edinburgh, Maclachlan and Stewart. 1840.

---

8. A History of the British Star-fishes and other Animals of the Class Echinodermata. By EDWARD FORBES, M.W.S. &c. Illustrated by Woodcuts and Vignettes. Part I. II. & III. VAN VOORST. October, November, and December, 1840. Price 2s. 6d. each.

---

9. A Probationary Essay on the Special Pathology of the Accessory Organs of Hearing. By JAMES MERCER, M.D. Licentiate of the Royal College of Surgeons, Edinb. &c. Pp. 133. Edinb. 1840.

---

10. Guy's Hospital Reports, No. XI. October, 1840. Edited by Drs. BARLOW and BABINGTON. S. Highley. Oct. 1840.

---

11. A Report of the Committee appointed by the Right Hon, the Governor of Bengal for the Establishment of a Fever Hospital, and for Inquiry into local Taxation in Calcutta. Quarto. Calcutta, 1839.

☞ *This is very creditable to a mixed Committee, in which the Majority were non-professional. We shall notice this Report shortly.*

---

12. Official Report of the Medical Topography and Climate of Calcutta, with brief Notices of its Prevalent Diseases, Endemic and Epidemic. By JAMES RANALD MARTIN, Presidency Surgeon, and Surgeon to the Native Hospital. Printed by Order of Government. Calcutta, 1839.

---

13. Practical Remarks on the Discrimination and Appearances of Surgical Disease; with an Appendix, containing the Descriptive Catalogue of the Author's Collection in Pathological Anatomy, and the Hunterian Oration for 1833. By JOHN HOWSHIP, Surgeon to the Charing Cross Hospital, &c. Octavo, pp. 420. Churchill, London. 1840.

---

14. The Anatomy of the Arteries of the Human Body; with its Application to Pathology and Operative Surgery in Lithographic Drawings. With Practical Commentaries. By RICHARD QUAIN, Professor of Anatomy in University College, and Surgeon to University College Hospital. The Delineations by JOSEPH MACLISE, Esq. Surgeon. Parts I. II. III. Price 12s. each. Taylor and Walton. Oct. 1840.

---

15. Outlines of Human Osteology. By P. O. WARD. Duodecimo, pp. 464. Renshaw. Oct. 1840. Part II.

---

16. State of an Institution near York, called the "RETREAT," for persons afflicted with Disorders of the Mind. By J. THURNAM, Resident Surgeon.

---

17. Provincial Medical and Surgical Journal. Edited by Dr. GREEN and Dr. STREETER. Nos. 1 and 2, for Oct. 3, and Oct 10, 1840. *In exchange.*

---

18. Human Physiology. By JOHN ELLIOTSON, M.D. &c. With which is incorporated much of the Elementary Part of the "Institutiones Physiologicæ" of J. F. BLUMENBACH, of Gottingen. Illustrated with numerous Woodcuts. Fifth Edition, pp. 1194. Longmans, 1840.

☞ *This splendid work is unequalled in the English, or, perhaps, in any other language, for variety of information, extent of research, and profundity of thought. We must notice it in another place. We grieve to see the plague-spot of mesmerism on the last two sheets of the work!!*

---

19. Vital Statistics of Scarborough. By JOHN LUNN, Surgeon. 4to, pp. 11. With several Tables. Simpkin and Marshall, 1840.

---

20. Elements of Chemistry, including the recent Discoveries and Doctrines of Science. By the late EDWARD TURNER, M.D. Sixth Edition, enlarged and revised, by JUSTUS LEIBEG, M.D. WILTON G. TURNER, M.D. and WM. GREGORY, M.D. Part III. No. 2. Price 5s. 6d. Taylor and Walton. London, October, 1840.

---

21. A Practical Treatise on Bilious Remittent Fever, its Causes and Effects. With illustrative Tables and Cases, &c. To which are added, Medical Topography of the different Military Stations in Jamaica. By W. ARNOLD, M.D. F.R.C.P. Edinb. &c. Octavo, pp. 320. Churchill, Oct. 1840.

---

22. A Practical Treatise on the Cure of Strabismus, or Squint, by Operation and by milder Treatment, with some new Views of the Anatomy and Physiology of the Muscles of the Eye. By P. BENNETT LUCAS, M.R.C.S. &c. Octavo, pp. 91, with colored Plates. Highley, 1840.

---

23. Operation for the Cure of Squinting: illustrated by explanatory Plates, the Drawings after Nature. Being an Appendix to a System of Practical Surgery. By JOHN LIZARS, Esq. &c. Pp. 7, with Plates. Edin. and Highley, London. 1840.

---

24. A Popular Treatise on Vaccination, its History, and present State, with regard to Re-vaccination. By WILLIAM REID, Surgeon. Octavo, pp. 40. Glasgow, D. Robertson. Edinb. Black and Co. Lond. Longman and Co.

---

25. Illustrations of Cutaneous Diseases. Part XXI. By ROBERT WILLIS, M.D. Price 5s. November, 1840.

---

26. The Pathology and Diagnosis of Diseases of the Chest, comprising a rational Exposition of their Physical Signs, with an Appendix, containing various Opinions and Experiments on the Motions and Sounds of the Heart, and on the Bronchi. By CHAS. J. B. WILLIAMS, M.D. F.R.S. &c. Fourth Edition, 8vo. pp. 322. Churchill, London, 1840.

---

27. Resolutions of the Cornwall Medical Association. 19th October, 1840.

---

28. Breviate of the Draft of the Medical Bill. By Mr. HAWES.

---

29. The History of the early and present State of the Venereal Disease examined; wherein is shewn that Mercury never was necessary for its Cure, &c. By G. HUME WEATHERHEAD, M.D. Octavo, pp. 258. Highley, Fleet Street. Nov. 1840.

---

30. Demonstrations of Anatomy: being a Guide to the Dissection of the Human Body. By GEO. VINER ELLIS, one of the Demonstrators of Anatomy in the University College. Octavo, pp. 620. Taylor and Co. Nov. 1840.

---

31. Essays and Heads of Lectures on Anatomy, Physiology, Pathology, and Surgery. By the late ALEX. MONRO, Secundus, M.D. Upwards of 50 years Professor of Anatomy and Surgery in the University of Edinburgh. With a Memoir of his Life by his Son and Successor. Octavo, pp. 132. with a Portrait and numerous Plates Maclachlan and Stewart, Edin. Nov. 1840.

---

32. The New York Journal of Medicine and Surgery, No. VI. Oct. 1840. *In exchange.*

---

33. Medico-Chirurgical Transactions, Second Series, Vol. V. Nov. 1840.

---

34. Illustrations of the Comparative Anatomy of the Nervous System. By Jos. Swan. Part VI. Price 7s. Longman and Co. Nov. 1840.

---

35. Thesis on the Nature and History of Plague, as observed in the North-western Provinces of India. By Fred. Forbes, M.D. Octavo, pp. 102. Maclachlan and Co. Edinb. 1840.

---

36. Elements of Chemistry, including the actual State and prevalent Doctrines of the Science, &c. By Ed. Turner, M.D., and W. Gregory, M.D. Octavo, pp. 998. Taylor and Walton. Dec. 1840.

---

37. Spinal Diseases: with an improved Plan of Cure, including what are commonly called Nervous Complaints. By John Hey Robertson, M.D. Glasgow, Dec. 1840. D. Robertson, Publisher.

---

28. Outlines of Medical Jurisprudence. (Course of Lectures.) By Thos. Stewart Trail, M.D. Octavo, pp. 222. Edinburgh, 1840. Second Edition. Black & Co.

---

39. Practical Observations on the Pathology and Treatment of Stricture of the Urethra, with Cases. By Robert Wade, Sen. Surgeon of the Westminster General Dispensary, &c. Octavo, pp. 149. Churchill, London. Dec. 1840.

---

40. A Treatise on the Nervous Diseases of Women; comprising an Inquiry into the Nature, Causes, and Treatment of Spinal and Hysterical Disorders. By Thomas Laycock, M.D. Octavo, pp. 356. Longman and Co. Dec. 1840.

---

41. An Address to the Medical Practitioners of Ireland on the Subject of Vaccination. Second Edition, illustrated by a Plate, with an Appendix, &c. By Samuel B. Labatt, M.D. Dublin, Hodges and Smith. Octavo, pp. 202. Dec. 1840.

---

42. Practical Remarks on the New Operation for the Cure of Strabismus or Squinting. Illustrated with Lithographic Engravings. By Edw. W. Duffin, Graduate of the University of Edinburgh, &c. Pp. 147. Churchill, London. Dec. 1840.

---

43. Introductory Lecture on Surgery, delivered on the 4th November, 1840. By James Miller, F.R.C.S.E., one of the Surgeons to the Royal Infirmary, Edinb.

---

44. A Discourse on the Phenomena of Sensation, as connected with the Mental, Physieal, and Instinctive Faculties of Man. By James Johnstone, M.D. Physician to the General Hospital of Birmingham, &c. Pp. 264. Churchill, London. Dec. 1840.

---

45. An Atlas of Plates, illustrative of the Principles and Practice of Obstetric Medicine and Surgery. With descriptive Letterpress. Nos. X. XI. and XII. By Francis H. Ramsbottom, M.D. &c. Churchill, 1840.

---

46. A Treatise on Stricture of the Urethra, with an Appendix on Dilatation by Fluid Pressure, &c. By James Arnott, M.D. Second Edition. Octavo, pp. 234. Sherwood and Co. 1840.

---

47. An Inquiry into the Efficacy of Digitalis in the Treatment of Idiopathic Epilepsy. By Edmund Sharkey, M.D. Lecturer on Midwifery, &c. Octavo, pp. 80. Highley, Dec. 1840.

---

44. A Treatise on the Sympathetic Relation between the Stomach and the Brain, and, throughout, between the Digestive Organs and the Nervous Systems, in the Causation and Cure of Disease. With an Appendix, &c. By Charles Wightman, M.D. Resident Physician at Newcastle-upon-Tyne, Octavo, pp. 192. Simpkin and Co. London, Dec. 1840.

---

49. On Scientific Medicine, and its Relations to, and Claims upon, Society at large. An Address read before the North of England Medical Association. By William Elliott, M.D. With an Appendix. Highley, Fleet Street. Price 1s. 6d. Dec. 1849.

☞ *A very clever Address.*

---

50. Malta: considered with reference to its Eligibility as a Place of Residence for Invalids. By Francis Sankey, M.D. Pp. 23.

51. Elements of Chemistry, &c. By ROBT. KANE, M.D. Professor of Natural Philosophy to the Royal Dublin Society, &c. Part I. Illustrated by 120 Woodcuts. Dublin, Hodges and Co. Dec. 1840.

52. Descriptive Catalogue of the Preparations in the Museum of the Royal College of Surgeons of Ireland. By JOHN HOUSTON, M.D. &c. Curator of the Museum, &c. Octavo, pp. 604. Hodges and Smith, Dublin; Renshaw, London. 1840.

☞ *This is a most valuable volume for those who have access to the collection—and indeed to every person, on account of the number of concise histories and cases appended to the descriptions. To be noticed farther in our next.*

53. Reasons for establishing a Sea-bathing Infirmary on the Western Coast, for the Benefit of the Poor, &c. By J. K. WALKER, M.D. Senior Physician to the Huddersfield Infirmary. Huddersfield, Dec. 1840.

☞ *We wish this proposal every degree of success.*

54. The Medical Profession, as it was, as it is, and as it ought to be. A Lecture introductory to the Business of the Original School of Medicine, Peter Street, Dublin, delivered by G. F. HAYDEN, A.B. M.B. T.C.D. &c. Octavo, pp. 24. Fannin and Co. Dublin. Dec. 1840.

55. Anatomie Pathologique du Corps Humain. Par M. CRUVEILHIER, M.D. Livraison No. 36. Bailliere, 1840.

56. Practical Hints on the Cure of Squinting by Operation. By F. W. GRANT CALDER, Assistant Surgeon to the Second Regiment of Life Guards. Octavo, pp. 96. Renshaw, Dec. 1840.

57. Memoranda regarding the Royal Lunatic Asylum, Infirmary, and Dispensary of Montrose, &c. By RICHARD POOLE, M.D. &c. Medical Superintendent of the Asylum. Octavo, pp. 218. Appendix, pp. 69. Longman and Co. 1841.

58. On the Diseases of the Hip-joint; with Observations on Affections of the Joints in the Puerperal State. With Plates. By WILLIAM COULSON, Surgeon to the Magdalen Hospital, &c. Second Edition, with Alterations and Additions. Longman and Co. 1841.

59. History of British Birds. By WM. YARRELL, F.L.S. &c. Parts XX. and XXI. VAN VOORST. London, September and November. 1840.

60. A General Outline of the Animal Kingdom, and Manual of Comparative Anatomy. By THOMAS RYMER JONES, F.Z.S. Prof. of Comparative Anatomy in King's College, London. With numerous Engravings on Wood. Van Voorst, London. Parts XII. and XIII. August and Decem. 1840. Price 2s. 6d.

### Notices to Correspondents.

*Ærated Magnesia Water.*—Mr. Pitt, of King Street, Snow Hill, has prepared a most agreeable beverage from Murray's "fluid magnesia," which is as brisk as soda water, and contains a considerable quantity of free carbonate of soda in the pint bottle. It forms a most delicious draught, extremely useful as an aperient and antacid.

*A regular Subscriber.*—Probably Dr. Blundell.

*Preparing for the Press.*—A new and greatly enlarged Edition of the "INFLUENCE of TROPICAL CLIMATES on EUROPEAN CONSTITUTIONS." By James Johnson, M.D. and James R. Martin, Esq. late Presidency Surgeon, and Surgeon to the Native Hospital, Calcutta.

⁂ *Mr. Martin will bring down all Information on Indian diseases and Topography to the present period in this the Sixth Edition.*

*Ceylon Moss.*—Mr. Previté has contrived to convert this nutritious substance into several agreeable forms, to suit the tastes and appetites of invalids. We have been trying and prescribing the jellies and the lozenges, of late, and can speak strongly in their favour. They can now be obtained at Savory and Moore's, and, we believe, at all the most respectable chemists in London.

*Erratum.*—By some strange mistake, the name of Wells was substituted for Walshe, in our notice of the article "Cancer," in the Cyclopædia of Practical Surgery. Dr. Walshe, of Camden Town, is the author of the article in question.

